# High-intensity interval training and β-hydroxy-β-methylbutyric free acid improves aerobic power and metabolic thresholds

**DOI:** 10.1186/1550-2783-11-16

**Published:** 2014-04-26

**Authors:** Edward H Robinson, Jeffrey R Stout, Amelia A Miramonti, David H Fukuda, Ran Wang, Jeremy R Townsend, Gerald T Mangine, Maren S Fragala, Jay R Hoffman

**Affiliations:** 1 Institute of Exercise Physiology & Wellness, University of Central Florida, 4000, Orlando, FL 32816, USA; 2 College of Education & Human Performance, 12494 University Boulevard, Orlando, FL 32816, USA

**Keywords:** High-intensity interval training, β-Hydroxy-β-Methylbutyric free acid, Aerobic, Endurance, *V*O_2_peak, Respiratory compensation point, Ventilatory threshold

## Abstract

**Background:**

Previous research combining Calcium β-hydroxy-β-methylbutyrate (CaHMB) and running high-intensity interval training (HIIT) have shown positive effects on aerobic performance measures. The purpose of this study was to examine the effect of β-hydroxy-β-methylbutyric free acid (HMBFA) and cycle ergometry HIIT on maximal oxygen consumption (*V*O_2_peak), ventilatory threshold (VT), respiratory compensation point (RCP) and time to exhaustion (T_max_) in college-aged men and women.

**Methods:**

Thirty-four healthy men and women (Age: 22.7 ± 3.1 yrs ; VO2peak: 39.3 ± 5.0 ml · kg^-1^ · min^-1^) volunteered to participate in this double-blind, placebo-controlled design study. All participants completed a series of tests prior to and following treatment. A peak oxygen consumption test was performed on a cycle ergometer to assess *V*O_2_peak, T_max_, VT, and RCP. Twenty-six participants were randomly assigned into either a placebo (PLA-HIIT) or 3 g per day of HMBFA (BetaTor™) (HMBFA-HIIT) group. Eight participants served as controls (CTL). Participants in the HIIT groups completed 12 HIIT (80-120% maximal workload) exercise sessions consisting of 5–6 bouts of a 2:1 minute cycling work to rest ratio protocol over a four-week period. Body composition was measured with dual energy x-ray absorptiometry (DEXA). Outcomes were assessed by ANCOVA with posttest means adjusted for pretest differences.

**Results:**

The HMBFA-HIIT intervention showed significant (*p* < 0.05) gains in *V*O_2_peak, and VT, versus the CTL and PLA-HIIT group. Both PLA-HIIT and HMBFA-HIIT treatment groups demonstrated significant (*p* < 0.05) improvement over CTL for T_max_, and RCP with no significant difference between the treatment groups. There were no significant differences observed for any measures of body composition. An independent-samples t-test confirmed that there were no significant differences between the training volumes for the PLA-HIIT and HMBFA-HIIT groups.

**Conclusions:**

Our findings support the use of HIIT in combination with HMBFA to improve aerobic fitness in college age men and women. These data suggest that the addition of HMBFA supplementation may result in greater changes in *V*O_2_peak and VT than HIIT alone.

**Study registration:**

The study was registered on ClinicalTrials.gov (ID NCT01941368).

## Background

High-intensity interval training (HIIT) has become a popular training modality in competitive athletes, recreationally-trained individuals, and clinical populations [1]. HIIT consists of repeated bouts of short to moderate duration exercise completed at intensities greater than the anaerobic threshold, interspersed with brief periods of low intensity or passive rest. The salient features of HIIT over constant rate aerobic training (CRT) are shorter training periods and the reported improvements of both oxidative and glycolytic energy systems [1,2].

Physiological adaptations associated with HIIT include improved metabolic efficiency [3,4] related to a more efficient skeletal muscle substrate utilization, and improved respiratory control sensitivity resulting from increased mitochondrial density [5]. Helgerud et al. [6] reported that an eight-week running HIIT program improved *V*O_2_max and time to exhaustion (TTE) more than CRT in moderately trained males. Further, Smith et al. [7] reported a 7% to 10% increase in *V*O_2_peak and ventilatory threshold (VT) values after only 3 weeks of HIIT on a cycle ergometer using college age males. HIIT, therefore, is a time-efficient exercise strategy that has shown consistent positive results following relatively short duration training programs.

The branched-chain amino acid, leucine, has shown to be the key contributor for muscle protein synthesis and may play a role as a substrate during this process [8]. As such, dietary supplementation of leucine and its metabolites has been demonstrated to provide anabolic or anti-catabolic effects on lean body mass during training or periods of energy imbalance [9-11]. Ingestion of one of these metabolites, β-hydroxy-β-methylbutyrate in the free acid form (HMBFA), has been suggested to provide similar benefits to those of leucine with regard to muscle protein synthesis [12]. Additional investigation with CaHMB and resistance training in humans has shown improvement in muscle mass and strength in both younger and older subjects [13-16]. Recently, scientists have suggested CaHMB may enhance the benefits of intense aerobic training by attenuating skeletal muscle damage and accelerating recovery between training bouts. In support, Knitter et al. [17] examined the effect of three grams of CaHMB or placebo per day in trained endurance athletes for six weeks. Following the training and supplementation period, blood markers of muscle damage, creatine phosphokinase (CPK) and lactate dehydrogenase (LDH), were measured in response to a 20-km race. Following the race, LDH and CPK levels were 10.5% and 17% lower in the CaHMB supplemented group, respectively compared to the placebo group. These results [17] suggest that CaHMB supplementation may attenuate some of the muscle damage often observed with endurance training, possibly reducing the incidence of overtraining and allowing for greater training adaptations.

Ingestion of CaHMB during an aerobic training program appears to provide additional benefits. Vukovich and Dreifort [18] examined the effect of 3 grams of CaHMB or placebo per day for 14 days in elite cyclists while average training volume was 300 miles per week. In response to only the CaHMB condition, the cyclists demonstrated a significant increase in peak oxygen consumption rate (VO_2_peak) and an increase in the onset of blood lactate accumulation during a graded exercise test. Those investigators suggested that changes in maximal and submaximal performance following CaHMB supplementation may have been related to both the attenuation of protein breakdown and the augmentation of mitochondrial protein synthesis resulting in greater oxidative energy capacity. In further support, Lamboley et al. [19] examined the effect of 5 weeks of CaHMB supplementation and HIIT in physically-active college students. They measured changes in *V*O_2_max, VT and respiratory compensation point (RCP) during a graded exercise test at baseline and post training. The HIIT running program was performed 3 times per week on a treadmill (1% grade) and participants supplemented with 3 grams per day of CaHMB or placebo. The results of the investigation demonstrated significant increases in *V*O_2_max, VT and RCP in both treatment groups from the HIIT; however, the CaHMB group resulted in a 19% to 45% greater increase in all metabolic variables. The investigators’ suggested that CaHMB may have attenuated the muscle damage often observed from running and might have accelerated recovery between training bouts. Further, CaHMB supplementation may have enhanced the training stimulus of HIIT on VT and RCP by increasing mitochondrial biogenesis, thus improving oxidative energy capacity and efficiency [13,18,19].

It appears that HMBFA supplementation is most effective during muscle damaging exercise [20]. Lamboley et al. [19] indicated that they specifically selected running to induce delayed onset muscle soreness, a non-invasive indicator of muscle damage. However, to date, no one has examined the effect of HMBFA supplementation while undergoing a HIIT program on a cycle ergometer. If muscle damage is needed to observe the potential benefits of HMBFA supplementation, then HIIT training on a cycle ergometer, which produces much less muscle damage [21] than running, may provide no additional benefit. Therefore, the purpose of this study was to examine the effects of chronic (4-weeks) HMBFA supplementation in combination with cycle ergometry HIIT on endurance performance measures in active college age men and women.

## Methods

### Participants

For inclusion in the study, all males were required to have a *V*O_2_peak greater than 35 ml∙kg^-1^∙min^-1^ and all female participants greater than 30 ml∙kg^-1^∙min^-1^. After initial testing, forty recreationally-active individuals (men = 21, women = 19) between the ages of 18 and 35 were recruited to participate in this study. Three female and two male participants were removed due to health reasons not associated with the study. One female participant was removed after a family emergency. Therefore, data for 19 men and 15 women (Table 1) were included in the final analysis. All participants completed a questionnaire to assess ability to participate in physical activity and to ascertain any prior supplementation regime. Individuals self-reported to be free of musculoskeletal injury as determined by a physical activity readiness questionnaire (PAR-Q). Following an explanation of all procedures, risks and benefits, each participant provided his/her informed consent to participate in the study.

**Table 1:** Participant descriptive statistics

**Variable**	**Control ** **(n = 8)**	**PLA-HIIT ** **(n = 13)**	**HMBFA-HIIT ** **(n = 13)**	**p-value**
Age (y)	21.0 ± 2.4	23.6 ± 3.7	22.9 ± 2.4	0.166
Height (cm)	171.4 ± 5.7	172.6 ± 12.2	173.0 ± 9.2	0.939
Body mass (kg)	76.3 ± 12.8	74.9 ± 16.6	72.4 ± 9.9	0.793
% Body fat	22.4 ± 8.1	19.7 ± 8.6	24.8 ± 8.1	0.301
Training volume (kJ)	N/A	1437.0 ± 309.6	1456.8 ± 378.6	0.313

Values are presented as means ± SD. HIIT, high-intensity interval training. HMBFA, β-hydroxy-β-methylbutyrate in the free acid form (BetaTor™, Metabolic Technologies Inc, Ames, IA), PLA, placebo.

A minimum sample size of n = 8 per group was determined using previously published data [19] and the formula derived by Gravettier and Wallnau [22] to achieve a statistical power (1-β) of 0.80. Therefore, with an expectation of subject dropout, a final sample size of n = 15 in each experimental group and n = 10 in the control group were recruited. The study was registered on ClinicalTrials.gov (ID NCT01941368).

### Research design

A double-blind, placebo-controlled design, stratified for gender, was used to examine the effects of HMBFA and HIIT training on measures of metabolic performance. Each participant was required to visit the Human Performance Laboratory on four separate occasions for pre- and post- testing, with each testing session occurring on nonconsecutive days. The same testing protocols were repeated at the beginning and end of the 4-week training period. On the first testing day, anthropometric measures of participants were collected (Table 1). Each participant then performed a graded exercise test to determine peak oxygen consumption (*V*O_2_peak), time to exhaustion (T_max_), respiratory compensation point (RCP), and ventilatory threshold (VT). The peak wattage achieved during this test was used to establish individual training intensity. On the second day of testing, a baseline blood draw was performed to measure serum HMB, and total lean soft tissue (TLST) and body fat percentage (BF) were assessed using dual energy x-ray absorptiometry (DEXA) (Prodigy™; Lunar Corporation, Madison, WI, USA).

After baseline testing, the participants were randomly assigned to one of three groups: a control group (CTL), a placebo with HIIT group (PLA-HIIT) or HMBFA with HIIT group (HMBFA-HIIT). Of the 40 subjects that were recruited for this study, 10 subjects were assigned to CTL and 15 to each of the training groups (PLA-HIIT or HMBFA-HIIT).

### Exercise protocol

**Figure 1:**
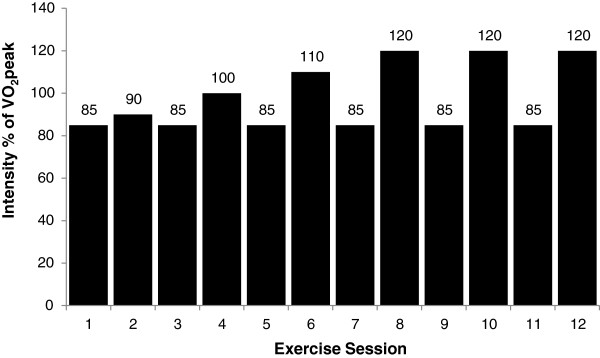
Exercise training intensity protocol.

Participants in the PLA-HIIT and HMBFA-HIIT groups participated in 4-weeks of high-intensity interval training with three sessions per week—with at least one day between each training session—on a calibrated, electronically-braked cycle ergometer (Lode Corival 400, Groningen, the Netherlands). The exercise training program consisted of alternating training sessions of sub-maximal and supra-maximal workloads (Figure 1). Each participant’s training load was determined as a percentage of the peak power output (P_peak_) from the graded exercise test. Individuals began each training session with a 5-minute warm up at a self-selected wattage, followed by an exercise protocol of five 2-minute exercise bouts at a predetermined percentage of their power output at *V*O_2_peak. Between each exercise bout, the participant had 1 minute of complete rest. In the event that there was an inability to complete the entire 2-min exercise bout, the participant completed the 1-min rest period and attempted subsequent bouts. Total time completed and power output was recorded for each exercise session to calculate total training volume (Power output (Watts) × Total time = Training Volume). Participants assigned to CTL were asked to continue their normal activity pattern for 4 weeks before returning to undergo post-testing.

### Supplementation

The HMBFA supplement consisted of 1 gram of β-hydroxy-β-methylbutyrate in the free acid form (BetaTor™, Metabolic Technologies Inc, Ames, IA), reverse osmosis water, de-bittering agent, orange flavor, stevia extract, and potassium carbonate. Each serving of placebo contained 1 gram of polydextrose that was equivalent to β-hydroxy-β-methylbutyrate in the free acid, citric acid, corn syrup, stevia extract, de-bittering agent, and orange flavoring. Identical in appearance and taste, the HMBFA and PL treatments were produced and supplied by Metabolic Technologies Inc. (Ames, IA). Prior to the first training session, subjects were randomly assigned to receive either 3 g per day of HMBFA or a placebo divided equally into three servings, given 30 minutes prior to exercise and again 1 hour later and then a final 1 g dose 3 hours post exercise on training days. To ensure compliance, investigators watched as the subjects consumed the supplement prior to and immediately after each exercise session. On the non-training days, subjects were instructed to consume one packet with three separate meals throughout the day. Empty packets were presented to the investigators upon returning to the laboratory following non-training days.

### Blood measurements and HMB analysis

During testing days, resting blood samples were drawn following a 15-min equilibration period. These blood samples were obtained from an antecubital arm vein using a 20-gauge disposable needle equipped with a Vacutainer® tube holder (Becton Dickinson, Franklin Lakes, NJ) containing K_2_EDTA. Each participant’s blood samples were obtained at the same time of day during each testing session. The blood was centrifuged at 3,000 × g for 15 min along and the resulting plasma was placed into s 1.8-mL microcentrifuge tube and frozen at -80°C for later analysis.

Plasma HMB concentrations were analyzed by gas chromatography–mass spectrometry which was performed by Metabolic Technologies Inc. in a blinded fashion using methods previously described by Nissen et al. [23].

### Dietary analysis

Prior to training, participants were asked to complete a 3-day food log, to establish macronutrient content and average leucine intake. This diet was considered the participant’s standard diet and they were asked to maintain a similar regimen throughout the duration of the study. These data were entered into a software program (Food Works 13, The Nutrition Company; Long Valley, NJ) which provided calculation for daily leucine intake (g) and total calories (kcal).

### Determination of *V*O_2_peak, VT, and RCP

An incremental test to volitional exhaustion was performed on an electronically-braked cycle ergometer (Lode Excalibur Sport; Groningen, The Netherlands) to determine *V*O_2_peak and the P_peak_ in watts (W) at *V*O_2_peak. Prior to testing, each participant was fitted with a Polar Heart Watch system to record the participants’ heart rate (Polar Electro Inc., Lake Success, NY). Following the procedures described by Bergstrom et al. [24], participants were instructed to maintain a pedaling cadence of 70–75 revolutions per minute (RPM) at an initial workload of 75 W. The workload increased 25 W every two minutes until he or she was unable to maintain a cadence above 70 RPM for ~10s despite verbal encouragement, or volitional fatigue. Prior to each graded exercise test, open-circuit spirometry (TrueOne 2400® Metabolic Measurement System, Parvo Medics, Inc., Sandy, UT) was calibrated with room air and gases of known concentration, which was used to estimate *V*O_2_peak (ml∙kg^-1^∙min^-1^) by sampling and analyzing breath-by-breath expired gases. Oxygen (O_2_), carbon dioxide (CO_2_), ventilation (*V*_E_), and respiratory exchange ratio (RER)—were monitored continuously and expressed as 30-second averages [25]. *V*O_2_peak was determined to be the highest 30-s *V*O_2_ value during the test and coincided with at least two of the following three criteria: (a) 90% of age-predicted maximum heart rate; (b) respiratory exchange ratio > 1.1; and/or (c) a plateau of oxygen uptake (less than 150 mL/min increase in *V*O_2_ during the last 60 s of the test). The test-retest reliability for *V*O_2_peak was ICC = 0.96 (SEM = 1.4 ml^.^kg^.^min^-1^).

Ventilatory threshold (VT) and RCP were determined by common methods for determining gas exchange thresholds [26-29]. The VT was determined by plotting and determining the point of increase in the *V*_E_/*V*O_2_ versus *V*O_2_ curve as the *V*_E_/*V*CO_2_ versus *V*O_2_ curve remained constant or decreased [24,26]. The RCP as described by Beaver et al. [26] was identified using the V-Slope method by plotting the *V*_E_ versus *V*CO_2_. The VT and RCP were reported as the corresponding *V*O_2_ and power output in watts (PVT and PRCP). The test-retest reliability for VT and RCP was ICC = 0.97 (SEM 0.1 ml^.^kg^.^min^-1^) and 0.87 (SEM 0.2 ml^.^kg^.^min^-1^), respectively.

### Anthropometric measures

Body composition was estimated from a scan by DEXA (GE Medical Systems Lunar, Madison, WI, USA; software version 13.60.033) performed by a state licensed x-ray technician. Participants were positioned supine in the center of the platform and were scanned using the default scan mode for total body scanning. Measures for total lean soft tissue (LSTM) and fat mass were calculated by the system software (Encore 2011, software version 13.60.033). Body composition was analyzed using estimated body fat percentage (%BF) and total lean soft tissue mass (LSTM). The test-retest reliability for LSTM and% BF was ICC = 0.99 (SEM 0.4 kg) and 0.99 (SEM 0.8%BF), respectively.

### Statistical analyses

Statistical software (IBM SPSS Statistics for Windows, Version 21.0; Armonk, NY: IBM Corp) was used to perform all statistical analysis. Separate one-way analyses of covariance (ANCOVA) were used to analyze all dependent performance and metabolic variable data based on the recommendations of Huck and McLean [30]. The independent variable, group, included 3 levels: PLA-HIIT, HMBFA-HIIT, and CTL. The pretest and posttest values were used as the covariate and dependent variable, respectively. When appropriate, LSD post hoc pairwise comparisons were used to examine the differences among the groups. For effect size, the partial eta squared statistic was calculated, and according to Green et al. [31], 0.01, 0.06, and 0.14 were interpreted as small, medium, and large effect sizes, respectively. Two-way ANOVA (Time × treatment) was used to examine changes in plasma HMB concentration between PLA-HIIT and HMBFA-HIIT. Independent-samples t-tests’ were performed to compare total training volume, total energy and leucine intake for the PLA-HIIT and HMBFA-HIIT groups. An alpha of p < 0.05 was established a priori.

## Results

The pre- and post-intervention mean and standard deviations for all metabolic and performance measures (*V*O_2_peak, P_peak_, T_max_, RCP, PRCP, VT, and PVT) for all groups (CTL, PLA-HIIT, HMBFA-HIIT) are provided in Table 2. Table 3 provides the group mean and standard deviations for pre- to post-intervention body composition measures (BW, LSTM, and BF).

**Table 2:** Metabolic and performance measures for pre- and post-supplementation

	**Control (*n*** **= 8)**		**PLA-HIIT (*n*** **= 13)**		**HMBFA-HIIT (*n*** **= 13)**	
**Measure**	**Pretest**	**Posttest**	**Pretest**	**Posttest**	**Pretest**	**Posttest**
VO_2_peak (ml · kg^-1^ · min^-1^)	39.1 ± 4.5	38.9 ± 4.0	38.9 ± 3.4	40.3 ± 2.6	39.8 ± 6.7	42.7 ± 5.1
P_peak_ (W)	218.8 ± 41.7	215.6 ± 32.6	221.2 ± 46.6	236.5 ± 48.5	226.9 ± 56.3	246.2 ± 54.8
T_max_ (min)	12.5 ± 2.9	12.2 ± 2.3	13.0 ± 3.8	14.2 ± 3.7	13.6 ± 4.7	14.9 ± 4.5
RCP (ml · kg^-1^ · min^-1^)	30.5 ± 5.0	28.7 ± 2.7	29.3 ± 3.1	31.9 ± 2.2	32.2 ± 4.2	33.7 ± 3.8
PRCP (W)	175.3 ± 38.8	167.0 ± 24.8	168.4 ± 36.0	185.0 ± 33.5	182.6 ± 33.6	196.5 ± 35.1
VT (ml · kg^-1^ · min^-1^)	27.7 ± 3.3	27.2 ± 2.7	28.6 ± 3.1	29.0 ± 4.1	27.8 ± 4.8	31.7 ± 3.7
PVT (W)	156.3 ± 17.7	153.1 ± 28.2	159.6 ± 40.2	169.2 ± 37.0	161.5 ± 39.02	184.6 ± 37.6

Values are means ± SD. HIIT, high-intensity interval training; HMBFA, β-hydroxy-β-methylbutyrate in the free acid form (BetaTor™, Metabolic Technologies Inc, Ames, IA); PLA, placebo; VO_2_peak, peak oxygen uptake; P_peak_, peak power achieved; Tmax, time to exhaustion during graded exercise test; RCP, respiratory-compensation point; VT, ventilatory threshold.

**Table 3:** Body composition measures for pre- and post-supplementation

	**Control (*n*** **= 8)**		**PLA-HIIT (*n*** **= 13)**		**HMBFA-HIIT (*n*** **= 13)**	
**Measure**	**Pretest**	**Posttest**	**Pretest**	**Posttest**	**Pretest**	**Posttest**
Body weight (kg)	76.3 ± 12.8	75.5 ± 12.7	74.9 ± 16.6	75.2 ± 16.3	72.4 ± 9.9	72.5 ± 10.0
Lean soft tissue mass (kg)	56.5 ± 11.7	56.4 ± 10.7	58.4 ± 16.6	58.6 ± 16.6	52.2 ± 10.9	52.2 ± 10.9
Total body fat mass (kg)	15.9 ± 7.0	14.3 ± 8.4	13.3 ± 4.8	13.2 ± 4.6	16.9 ± 5.3	17.0 ± 5.4
Body fat %	22.4 ± 8.1	22.0 ± 2.8	19.7 ± 8.6	19.5 ± 8.4	24.8 ± 8.1	24.6 ± 7.7

Values are means ± SD. HIIT, high-intensity interval training; HMBFA, β-hydroxy-β-methylbutyrate in the free acid form (BetaTor™, Metabolic Technologies Inc, Ames, IA); PLA, placebo.

### Peak oxygen uptake (*V*O_2_peak)

**Figure 2:**
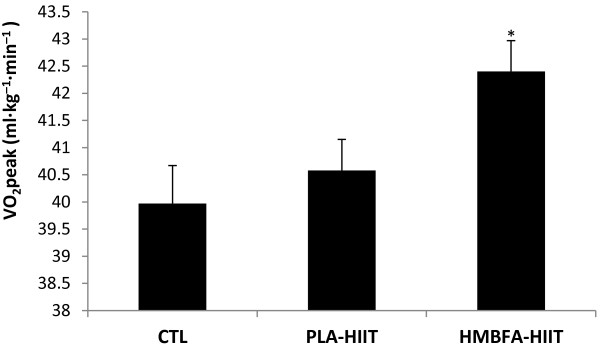
**VO**_**2**_**peak obtained during graded exercise test.** Mean values (+SEM) for posttest VO_2_peak scores adjusted for the initial differences in pretest VO_2_peak (covariate; adjusted pretest mean = 39.3). *HMBFA-HIIT significantly greater than PLA-HIIT (*p* = 0.032) and CTL (*p* = 0.001).

The ANCOVA indicated a significant difference (*p* = 0.003, η^2^ = 0.322) among the group means for the posttest *V*O_2_peak values after adjusting for pre-test differences (Figure 2). The strength of the association (i.e., effect size, η^2^) indicated that the treatment groups (CTL, PLA-HIIT, HMBFA-HIIT) accounted for 32% of the variance of the post-test *V*O_2_peak values, holding constant the pre-test *V*O_2_peak scores. The LSD pairwise comparisons indicated that the increase in *V*O_2_peak from pre- to post-testing was greater for the HMBFA-HIIT group than for the CTL (*p* = 0.001) and the PLA-HIIT groups (*p* = 0.032), however, no differences were found between PLA-HIIT and CTL groups (*p* = 0.09). The group means (±SEM) for the post-test *V*O_2_peak values, adjusted for initial differences in pre-test scores, are shown in Figure 2.

### Peak power (P_peak_)

**Figure 3:**
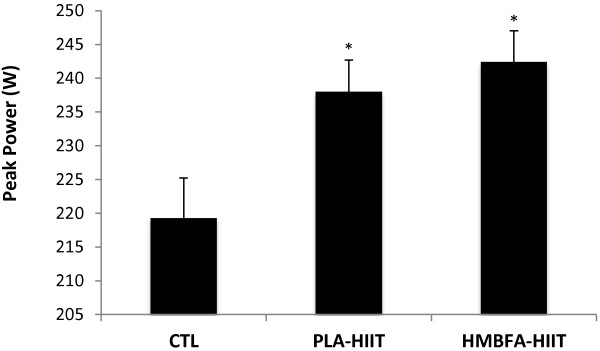
**Peak power (P**_**peak**_**) obtained during graded exercise test.** Mean values (+SEM) for posttest P_peak_ scores adjusted for the initial differences in pretest P_peak_ (covariate; adjusted pretest mean = 222.79). *Indicates significantly different than CTL (PLA-HIIT, p = 0.018; HMBFA-HIIT, *p* = 0.04).

The ANCOVA indicated a significant difference (*p* = 0.013, η^2^ = 0.251) among the group means for the post-test P_peak_ values after adjusting for pre-test differences (Figure 3). The strength of the association (i.e., effect size, η^2^) indicated that the treatment groups (CTL, PLA-HIIT, HMBFA-HIIT) accounted for 25% of the variance of the post-test P_peak_ values, holding constant the pre-test P_peak_ scores. The LSD pairwise comparisons indicated that the increase in P_peak_ from pre- to post-testing was greater for the HMBFA-HIIT (p = 0.04) and PLA-HIIT (*p* = 0.018) groups than for the CTL group, however, no differences were found between HMBFA-HIIT and PLA-HIIT groups (*p* = 0.51). The group means (±SEM) for the post-test P_peak_ values, adjusted for initial differences in pre-test scores, are shown in Figure 3.

### Time to exhaustion (T_max_)

**Figure 4:**
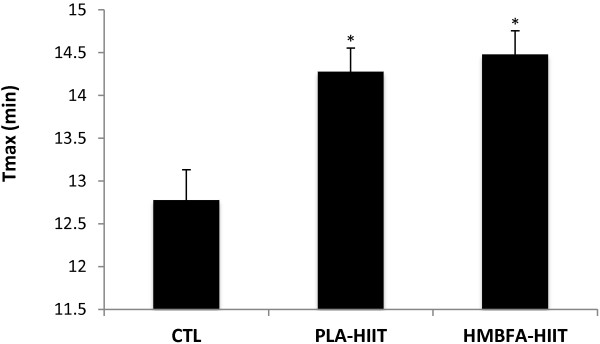
**Time to exhaustion (T**_**max**_**) for the graded exercise test.** Mean values (+SEM) for posttest T_max_ scores adjusted for the initial differences in pretest T_max_ (covariate; adjusted pretest mean = 13.11). *Indicates significantly different than CTL (PLA-HIIT, p = 0.002; HMBFA-HIIT, *p* = 0.001).

The ANCOVA indicated a significant difference (*p* = 0.002, η^2^ = 0.35) among the group means for the post-test T_max_ values after adjusting for pre-test differences (Figure 4). The strength of the association (i.e., effect size, η^2^) indicated that the treatment groups (CTL, PLA-HIIT, HMBFA-HIIT) accounted for 35% of the variance of the post-test T_max_ values, holding constant the pre-test T_max_ scores. The LSD pairwise comparisons indicated that the increase in T_max_ from pre- to post-testing was greater for the HMBFA-HIIT (*p* = 0.001) and PLA-HIIT (*p* = 0.002) groups than for the CTL group, however, no differences were found between HMBFA-HIIT and PLA-HIIT groups (*p* = 0.62). The group means (±SEM) for the post-test T_max_ values, adjusted for initial differences in pretest scores, are shown in Figure 4.

### Respiratory Compensation Point (RCP)

**Figure 5:**
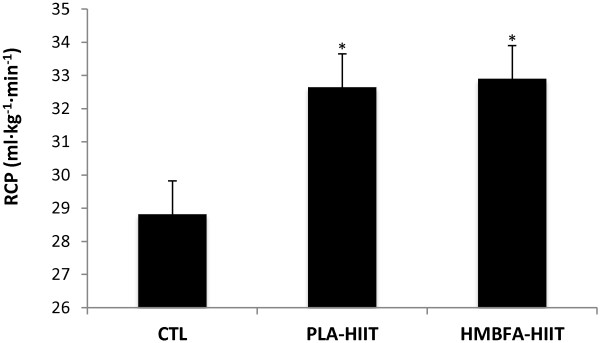
**Respiratory compensation point (RCP).** Mean values (+SEM) for posttest RCP scores adjusted for the initial differences in pretest RCP (covariate; adjusted pretest mean = 30.69). *Indicates significantly different than CTL (PLA-HIIT, p < 0.001; HMBFA-HIIT, p < 0.001).

The ANCOVA indicated a significant difference (*p* < 0.001, η^2^ = 0.436) among the group means for the posttest RCP values after adjusting for pre-test differences (Figure 5). The strength of the association (i.e., effect size, η^2^) indicated that the treatment groups (CTL, PLA-HIIT, HMBFA-HIIT) accounted for 44% of the variance of the post-test RCP values, holding constant the pre-test RCP scores. The LSD pairwise comparisons indicated that the increase in RCP from pre- to post-testing was greater for the HMBFA-HIIT (*p* < 0.001) and PLA-HIIT (*p* < 0.001) groups than for the CTL group, however, no differences were found between HMBFA-HIIT and PLA-HIIT groups (*p* = 0.77). The group means (±SEM) for the posttest RCP values, adjusted for initial differences in pretest scores, are shown in Figure 5.

### Power at Respiratory Compensation Point (PRCP)

**Figure 6:**
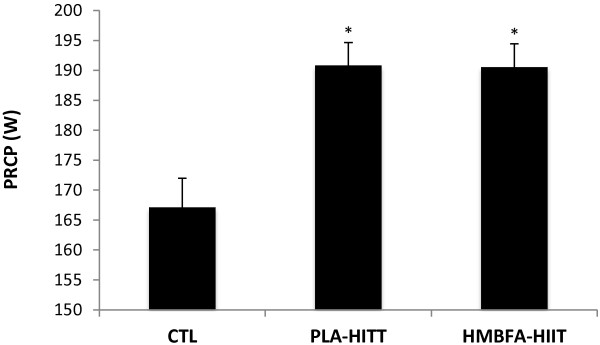
**Power at respiratory compensation point (PRCP).** Mean values (+SEM) for posttest PRCP scores adjusted for the initial differences in pretest PRCP (covariate; adjusted pretest mean = 175.43). *Indicates significantly different than CTL (PLA-HIIT, p = 0.001; HMBFA-HIIT, p = 0.001).

The ANCOVA indicated a significant difference (*p* = 0.001, η2 = 0.375) among the group means for the posttest PRCP values after adjusting for pre-test differences (Table 2, Figure 6). The strength of the association (i.e., effect size, η^2^) indicated that the treatment groups (CTL, PLA-HIIT, HMBFA-HIIT) accounted for 38% of the variance of the post-test PRCP values, holding constant the pre-test PRCP scores. The LSD pairwise comparisons indicated that the increase in PRCP from pre- to post-testing was greater for the HMBFA-HIIT (*p* < 0.001) and PLA-HIIT (*p* < 0.001) groups than for the CTL group, however, no differences were found between HMBFA-HIIT and PLA-HIIT groups (*p* = 0.97). The group means (±SEM) for the posttest PRCP values, adjusted for initial differences in pretest scores, are shown in Figure 6.

### Ventilatory Threshold (VT)

**Figure 7:**
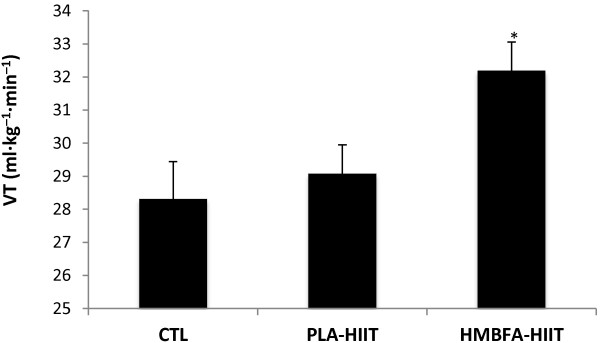
**Ventilatory Threshold (VT).** Mean values (+SEM) for posttest VT scores adjusted for the initial differences in pretest VT (covariate; adjusted pretest mean = 28.68). *HMBFA-HIIT significantly greater than PLA-HIIT (*p* = 0.017) and CTL (*p* = 0.012).

The ANCOVA indicated a significant difference (*p* = 0.016, η2 = 0.24) among the group means for the post-test VT values after adjusting for pre-test differences (Figure 7). The strength of the association (i.e., effect size, η^2^) indicated that the treatment groups (CTL, PLA-HIIT, HMBFA-HIIT) accounted for 24% of the variance of the posttest VT values, holding constant the pre-test VT scores. The LSD pairwise comparisons indicated that the increase in VT from pre- to post-testing was greater for the HMBFA-HIIT group than for the CTL (*p* = 0.012) and the PLA-HIIT groups (*p* = 0.017), however, no differences were found between PLA-HIIT and CTL groups (*p* = 0.6). The group means (±SEM) for the posttest VT values, adjusted for initial differences in pretest scores, are shown in Figure 7.

### Power at Ventilatory Threshold (PVT)

**Figure 8:**
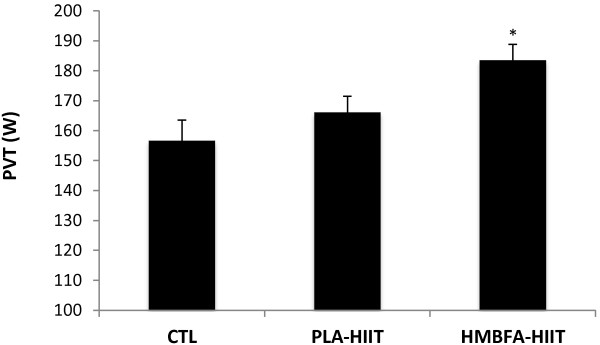
**Power at ventilatory threshold (PVT).** Mean values (+SEM) for posttest PVT scores adjusted for the initial differences in pretest PVT (covariate; adjusted pretest mean = 160.29). *HMBFA-HIIT significantly greater than PLA-HIIT (*p* = 0.027) and CTL (*p* = 0.004).

The ANCOVA indicated a significant difference (*p* = 0.009, η^2^ = 0.267) among the group means for the post-test PVT values after adjusting for pre-test differences (Figure 8). The strength of the association (i.e., effect size, η^2^) indicated that the treatment groups (CTL, PLA-HIIT, HMBFA-HIIT) accounted for 27% of the variance of the post-test PVT values, holding constant the pre-test PVT scores. The LSD pairwise comparisons indicated that the increase in PVT from pre- to post-testing was greater for the HMBFA-HIIT group than for the CTL (*p* = 0.004) and the PLA-HIIT groups (*p* = 0.027), however, no differences were found between PLA-HIIT and CTL groups (*p* = 0.277). The group means (±SEM) for the posttest PVT values, adjusted for initial differences in pretest scores, are shown in Figure 8.

### Body composition

The ANCOVA indicated no significant difference for body mass (*p* = 0.31, η^2^ = 0.074) percent body fat (*p* = 0.88, η^2^ = 0.009), and lean soft tissue mass (*p* = 0.247, η^2^ = 0.089) between the groups (Table 3).

### Training volume

There was no significant difference (*p* = 0.31) between training volumes for PLA-HIIT (1437.0 ± 309.6 kJ) and HMBFA-HIIT (1456.8 ± 378.6 kJ).

### Dietary analysis

There was no significant difference for daily energy intake (*p* = 0.159; PLA-HIIT, 2398.7 ± 619 Kcal; HMBFA-HIIT, 2011 ± 620 Kcal) or leucine intake (*p* = 0.561; PLA-HIIT, 3.3 ± 1.7 g; HMBFA-HIIT, 3.9 ± 2.1 g) between the two treatment groups.

### Supplementation compliance and plasma HMBFA concentrations

Placebo or HMBFA intake was recorded on individual intake logs, which were returned to the laboratory and monitored and resulted in 99% compliance. In addition, there was a significant interaction (F = 5.9, p = 0.02) for blood plasma HMBFA concentrations. The HMBFA-HIIT group increased by 2.6 ± 2.1 nmol∙ml^-1^ with little change in the PLA-HIIT group (0.1 ± 0.9 nmol∙ml^-1^), further supporting compliance in the treatment group.

## Discussion

To the best of our knowledge, this is the first study to examine the effects of the free-acid form of β-hydroxy-β-methylbutyrate (HMB) supplementation and high-intensity interval training (HIIT) using a cycle ergometer on metabolic and performance measures in young men and women. The primary findings support the use of HIIT in combination HMBFA as a training method to improve aerobic fitness. Furthermore, the results of the current study suggest that HMBFA supplementation significantly improved the benefits of the 4-week HIIT program on VO2peak, VT and PVT aerobic and metabolic measures when compared to HIIT alone.

The HIIT protocol used in the current study (Figure 1) resulted in a 4 to 11% increase in aerobic performance measures (*V*O_2_peak, P_peak_, T_max_; Table 2). This is consistent with Smith et al. [7] who reported a 7% to 11% increase in *V*O_2_peak and T_max_ after 3 weeks of HIIT using a similar protocol. In agreement, several other studies have reported 7 to 10% increases in *V*O_2_peak using HIIT protocols in college-aged participants [6,32,33]. Although previous studies utilizing this method of HIIT utilized a 5-day per week training routine, Jourkesh et al. [34] also reported a significant increase in T_max_ after 3 weeks of periodized HIIT and a significant increase in *V*O_2_peak after 6 weeks with training 3 times per week.

In the current investigation, the addition of HMBFA ingestion with HIIT significantly (7.3%) increased *V*O_2_peak (Table 2, Figure 2) greater than training alone. The present results are in agreement with Lamboley et al. [19] who reported a 15% increase in *V*O_2_max after 5 weeks of a running HIIT program while supplementing with 3 grams per day of calcium β-hydroxy-β-methylbutyrate (CaHMB) in college age men and women. In contrast, previous studies, which involved supplementation of CaHMB while endurance training, found no increase in *V*O_2_peak with 2 to 6 weeks of supplementation [17,18]. In a cross-over design, Vukovich and Dreifort [18], examined the effect of CaHMB supplementation in endurance-trained cyclists, and reported no significant increase in *V*O_2_peak in these highly trained athletes, however, there was a significant increase (3.6%) in the time to reach *V*O_2_peak (T_max_). The increase in T_max_ observed by Vukovich and Dreifort [18], was smaller than our observed 8% increase in younger untrained men and women (Table 2). The discrepancy between our study and the previous endurance training studies [18] examining CaHMB could be due to the training experience of the participants used in the investigation. It has been suggested that active men and women who are unaccustomed to HIIT may benefit more from CaHMB supplementation than trained athletes who are accustomed to HIIT [19]. The participants in the current study were unfamiliar with HIIT, which may explain why our results were similar to Lamboley et al. [19] and not Vukovich et al. [18] who used trained endurance athletes. However, Knitter et al. [17] also examined individuals unaccustomed to HIIT, but reported no additive effect of HMB supplement on endurance performance measures. Therefore, training status and previous experience with HIIT could have influenced the current results while explaining differences from previous investigations.

The differences reported by Lamboley et al. [19] and our findings versus other studies may be due to the fact that individualized HIIT programs were developed based on each participant’s baseline fitness level and monitored throughout the 28 days of training, while it was unclear what endurance program was used in other studies [18]. Therefore, the difference in results by Knitter [17] and Vukovich et al. [18] in comparison to Lamboley et al. [19] and our data may be related to an insufficient training stimulus that was unable to stimulate physiological adaptation [13,20,35].

Fatigue threshold measures, such as VT, RCP, and onset of blood lactate accumulation (OBLA), have been used as non-invasive measures of health and performance, and in the evaluation of the efficacy of endurance training and/or nutritional supplementation [19,36,37]. Further, the measurement of specific fatigue thresholds during a graded exercise test, like VT and RCP, may be useful for demarcating the heavy or severe exercise intensity domains, respectively [24]. For example, VT has been associated with the minimum exercise intensity that results in excessive CO_2_ production from the bicarbonate buffering of hydrogen ions [38,39], while exercise above RCP has been associated with the severe intensity domain which leads to excessive minute ventilation resulting from hyperkalemia [24,40]. The measurement of fatigue thresholds (VT, RCP), therefore, may provide possible mechanistic explanation for aerobic performance changes from training or nutritional interventions. Additionally, assessment of the exercise intensity domains, heavy (VT), severe (RCP) and maximal (*V*O_2_peak), during a graded exercise test may improve the sensitivity of detecting the potential effects on aerobic performance from various exercise and or nutritional interventions due to different mechanisms.

In the current study, the four-week HIIT program resulted in a 6.3% increase in power output at ventilatory threshold (PVT) (Table 2) which is similar to Smith et al. [7] who reported a ~9% increase using a comparable three-week HIIT cycling protocol in untrained college aged men. In addition, our study demonstrated an 8.6% increase in RCP which was very similar to the changes reported by Lamboley et al. [19] of an 8.5% increase from 5 weeks of HIIT on a treadmill. Our data, along with Smith et al. [7] and Lamboley et al. [19], support previous studies that demonstrate HIIT consistently improves metabolic threshold measures [6,41,42].

The addition of HMBFA to the four weeks of HIIT (HMB-HIIT) resulted in a ~14% increase in VT which was significantly greater than HIIT alone (Table 2, Figure 7). However, there was no difference between HMBFA-HIIT and PLA-HIIT groups for changes in RCP (Table 2, Figure 5). While our changes in VT are similar to values reported by Lamboley et al. [19], they described no significant difference between CaHMB-HIIT and PLA-HIIT groups. However, Lamboley et al. [19] reported significantly greater changes in RCP for CaHMB-HIIT compared to PLA-HIIT, whereas the current investigation resulted in similar changes between groups. Furthermore, Vukovich and Dreifort [18] reported a 9.1% increase in OBLA after two weeks of CaHMB supplementation in elite cyclists. Previous researchers have used OBLA as a method to identify the crossover point between moderate to heavy exercise intensities denoted by blood lactate concentrations greater than 4 mmol∙L^-1^ during an incremental exercise test [43]. With previous evidence supporting OBLA and VT as fatigue thresholds representing similar exercise domains, the increases in exercise intensity at OBLA (+9.1%) reported by Vukovich and Dreifort [18] and the increase in VT (+14%) observed in our study (Table 2) may reflect similar physiological adaptations. Our results, along with Vukovich and Dreifort [18] and Lamboley et al. [19], suggest that HMBFA may augment the beneficial effects of HIIT on aerobic performance by increasing fatigue threshold measures that reflect the physiological response to moderate and/or severe intensity exercise.

The physiological changes observed in aerobic performance from HIIT have been shown to improve *V*O_2_peak, muscle buffering capacity, and whole body fat oxidation [1,44,45]. Further, the improved aerobic power associated with HIIT has been linked to an up-regulation of glycolytic enzymes, as well as, increased mitochondrial density and blood flow [46,47]. HMBFA supplementation may improve HIIT training by up-regulating fatty acid oxidation, adenosine monophosphate kinase (AMPK), Sirt1, and Sirt3 activity in muscle cells [48,49]. Sirt1, Sirt3, and AMPK have been shown to augment mitochondrial biogenesis, lipolysis, energy metabolism and the reactive oxygen defense system [50,51]. Additionally, Pinheiro et al. [49] reported that 28 days of CaHMB administration in male Wistar rats resulted in significantly increased intramuscular ATP and glycogen content. While speculative, HMBFA supplementation may have enhanced the effects of HIIT by improving mitochondrial biogenesis, fat oxidation, and metabolism. However, more research is needed to support these proposed mechanisms in humans.

## Conclusions

In conclusion, our findings support the use of HIIT in combination with HMBFA as an effective training stimulus for improving aerobic performance. In addition, the use of HMBFA supplementation, in combination with HIIT, appeared to result in greater changes in *V*O_2_peak, PVT and VT than HIIT alone. While more research is needed, the current investigation suggests that in this sample of college age men and women, the use of HMBFA supplementation may enhance the benefits of HIIT on aerobic performance measures.

## Competing interests

The authors declare that they have no competing interests.

## Authors’ contributions

All authors contributed equally to this work. All authors have read and approved the final manuscript.

## Authors’ information

The authors responsible for this work are members of the Institute of Exercise Physiology and Wellness at the University of Central Florida. All authors are faculty and graduate students in the College of Education and Human Performance.
